# Allergy to Ringer’s Lactate - Uncommon Cause of Anaphylaxis during Intraoperative Period: A Case Report

**DOI:** 10.31729/jnma.v64i293.9287

**Published:** 2026-01-31

**Authors:** Shova Dangol, Surendra Man Shrestha, Prabhat Rawal, Nitendra Raj Bajracharya, Kundu Shrestha, Saurav Shrestha, Pushkar B.K., Rabi Paudel

**Affiliations:** 1 Department of Anesthesiology and Critical Care, Nepal Armed Police Force Hospital, Balambu, Kathmandu Nepal

**Keywords:** *anaphylaxis*, *intravenous fluids*, *perioperative allergy*, *ringer 's lactate*

## Abstract

Perioperative anaphylaxis is a rare but potentially fatal event, with an incidence ranging from 1:300 to 1:20,000 surgeries and a mortality rate of 3-9%. Its intraoperative occurrence is diagnostically challenging due to patient sedation and overlapping drug effects. Crystalloids like Ringer’s Lactate are rarely implicated. A 27-year-old American Society of Anesthesiologists grade I female undergoing mastoidectomy developed intraoperative hypotension, tachycardia, and generalized erythema after induction with standard anesthetics and Ringer’s Lactate infusion. Anaphylaxis was suspected, and Ringer’Lactate was discontinued. The patient improved rapidly upon switching to normal saline. A subcutaneous challenge postoperatively confirmed Ringer’s Lactate as the trigger. This case highlights an uncommon but serious hypersensitivity to Ringer’s Lactate. Clinicians must maintain high suspicion for all agents, including crystalloids, during Perioperative anaphylaxis. Prompt recognition, discontinuation of the offending agent, and supportive treatment are crucial. Given the widespread use of Ringer’s Lactate, awareness of this rare reaction is essential to prevent misdiagnosis and ensure patient safety.

## INTRODUCTION

Perioperative anaphylaxis is a rare and life-threatening condition.^1,2^ It is even more dangerous during an intraoperative period because of its diagnostic challenges, as the patient is not able to complain about the symptoms and cutaneous changes which are concealed by sterile drapes. Identifying the possible culprit is equally difficult as multiple agents are administered during anesthesia and surgery which have similar adverse effects as that of anaphylaxis. Hypotension and tachycardia can be caused by Propofol and Isoflurane given during induction and maintenance of anesthesia.

The reported incidence of perioperative anaphylaxis (POA) is 1 in 300 to 400 to 1 in 20,000 surgical procedures^2-5^ and its mortality rate is in the range of 3-9%. Brain injury secondary to anoxia is the most important morbidity and occurs in about 2% of patients affected by anaphylaxis.^6^

## PATIENT PROFILE

A 27-year-old female, weighing 57 kg and measuring 152 cm in height, with no significant medical and allergic history, was scheduled for a right modified radical mastoidectomy.

## PREOPERATIVE COURSE

Pre-anesthetic checkup was done a day before planned surgery and the patient was shifted to the preoperative room on the day of surgery. Intravenous cannulation was performed in the left hand using an 18G cannula. Ceftriaxone (1 gm IV) was administered after giving a test dose before skin incision. The patient was transferred to the operating room, where American Society of Anesthesiologists (ASA) standard I and II monitors were attached. Preoperative vital signs were as follows: blood pressure (BP) 110/60 mmHg, heart rate (HR) 60 bpm, SpO2 99% on room air, and respiratory rate 14/min.

## INDUCTION AND INTUBATION

Intravenous (IV) Ringer’s Lactate (RL) was initiated and 100% oxygen was administered. Anesthesia was induced with intravenous midazolam (2 mg), fentanyl (100 mcg), and a titrated dose of propofol to achieve loss of the eyelash reflex. Vecuronium (6 mg) was administered for muscle relaxation. Intubation was accomplished using a 7 mm internal diameter flexometallic endotracheal tube, secured at 20 cm. Ventilator settings included a tidal volume of 400 ml, respiratory rate of 12/min, an inspiratory-to-expiratory (I:E) ratio of 1:2, a PEEP of 5 cm H_2_O, and a maximum airway pressure (Pmax) of 30 cm H_2_O. Maintenance was done with Sevoflurane 2% with 2 liters/minute of oxygen.

## INTRAOPERATIVE EVENTS

Sterile draping was done and 5ml of undiluted 2% lignocaine with adrenaline was administered to the incision site by the operating surgeon. Just after the incision, patient’s BP dropped to 80/50 mmHg with Mean Arterial Pressure (MAP) 57 mmHg. IV Mephentermine 6mg was administered, raising the MAP to 65 mmHg. Subsequently, the patient’s HR increased to 130 bpm, and an additional dose of Fentanyl (20 mcg) and Vecuronium (1 mg) was given.

We attributed the increase in heart rate to local absorption of Adrenaline from the same initially. But the heart rate persistently rose above 130 bpm. The BP further decreased to 76/44 mmHg, necessitating another dose of mephentermine (6 mg). Concurrently, generalized erythematous rashes were observed in the left hand with IV cannulation, initially attributed to a possible ceftriaxone allergy. Antihistamine (IV Pheniramine 22.5mg) and Corticosteroid (IV Hydrocortisone, 100 mg) were administered. When examining all the body parts, we found rashes all over the body including chest, abdomen and legs. The peripheries were warm and the rashes were blanchable.

**Figure 1:**
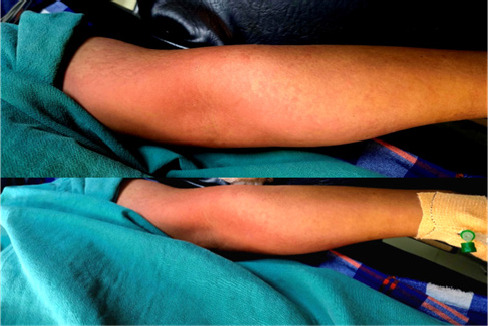
Erythematous rashes on the upper extremity of patient.

**Figure 2:**
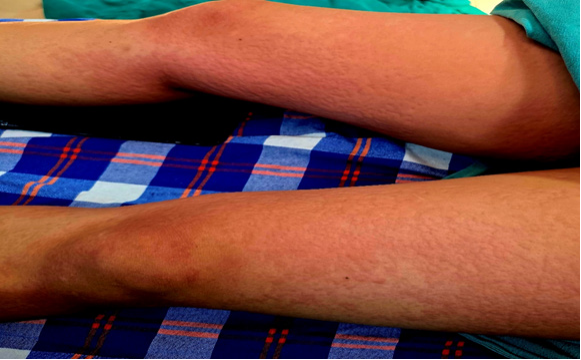
Erythematous rash on the lower extremity of patient.

**Figure 3:**
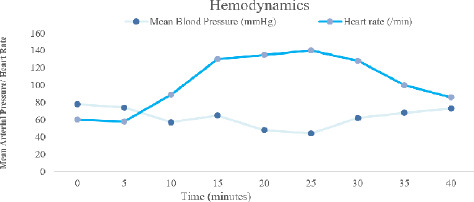
Patient’s Intraoperative Hemodynamics.

## ANAPHYLAXIS DIAGNOSIS AND MANAGEMENT

Despite initial treatment, the patient exhibited persistent hypotension (BP 81/39 mmHg) and tachycardia (HR 133 bpm). Phenylephrine (50 mcg) was given, temporarily stabilizing BP to 98/68 mmHg and HR to 110 bpm. Subsequent hypotensive episodes were managed with additional phenylephrine boluses. A drop in SpO2 to 90% prompted manual ventilation, improving saturation to 96%. Given the persistence of hypotension, tachycardia, and rash, with normal breath sounds, Ring and Messmer grade II anaphylaxis was suspected.^6,7^ Since one of the continuous agents administered to the patient was IV fluids, Ringer’s Lactate was discontinued, and IV fluids were switched to normal saline from a different pharmaceutical company. A second dose of hydrocortisone (100 mg IV) was administered. Within five minutes, the patient’s HR stabilized at 88 bpm, and BP maintained a MAP of 67-75 mmHg. The erythematous rash resolved completely after around an hour ofdiscontinuing Ringer’s Lactate. ABG was done and all parameters were within normal limits. Surgery was completed and the patient was shifted to the recovery room. To confirm the causative agent for anaphylaxis and the symptoms, 1 ml of Inj. Ringer’s lactate was given subcutaneously in post-operative room. There was development of rashes around the injection site and patient complained of itching which confirmed the anaphylaxis to Ringer’s Lactate.^8^

## DISCUSSION

Ringer’s lactate solution was developed in the 1880s by Sydney Ringer, a British physiologist. It was later modified by Alexis Hartmann in the 1930s by adding lactate to avoid acidosis especially in paediatrics cases.^9^ Hence the name, Hartmann’s solution. It is one of the most common IV fluids, used to replenish body fluids and electrolytes and maintain blood sugar level during perioperative period. Ringer’s lactate solution is generally considered safe and non-irritant. Anaphylaxis caused by RL is rarely reported. So far, only one case was reported where the attending anesthesiologist encountered anaphylaxis due to RL.^10^

In the case of our patient, she had no known history of previous allergies and anaphylaxis. However, she was concomitantly administered IV anesthetic drugs including fentanyl, propofol, vecuronium and antibiotics as ceftriaxone, her symptoms of anaphylaxis resolved only after stopping of IV Ringer’s Lactate. Immediate hypersensitivity to ceftriaxone was initially considered, as beta-lactam antibiotics are among the leading triggers of perioperative anaphylaxis. However, there was no reaction to the test dose and symptom onset was delayed relative to antibiotic administration. Further, hemodynamic instability persisted despite antihistamines and corticosteroids, resolving only after discontinuation of Ringer’s lactate indicating an unlikely role of ceftriaxone in triggering the hypersensitivity reaction.^11-12^

Opioid related adverse events, including fentanyl induced chest wall rigidity, were also evaluated. Such reactions typically occur soon after opioid administration and present with increased airway pressures and sometimes even impaired ventilation and thus, hypercapnia. In this case, airway pressure remained normal and oxygenation improved with supportive care.^11^

There is minimal chance of local anesthetic-induced systemic toxicity since lignocaine with adrenaline was given within recommended limits and there were no neurological features of systemic toxicity. Moreover, the progression of hypotension with generalized edema is not characteristic of local anesthetic reactions.^13^

Although vasodilation from anesthetic agents such as propofol or volatile anesthetics is a frequent cause of perioperative hypotension, this usually occurs without cutaneous manifestations or persistent tachycardia.^14^

The combination ofrecurrent hypotension, sustained tachycardia, warm peripheries and generalized blanchable erythema instead supports a systemic hypersensitivity reaction. The cardiovascular instability, diffuse cutaneous involvement and rapid sustained improvement after cessation of Ringer’s lactate are most consistent with Grade II perioperative anaphylaxis according to Ring and Messmer classification.^12^

The exact cause of such reactions is unclear, though some suggest that additives like di-2-ethylhexyl phthalate (DEHP), used in the solution’s packaging, might contribute. Studies on DEHP’s impact on immune responses have shown heightened reactions at lower doses, which could explain allergic symptoms even after small volumes of infusion.^10^

Diagnostic approaches for drug allergies, including skin tests and serum antibody measurements, have limited sensitivity for colloids like Ringer’s Lactate.^15^ In this case, the patient declined further testing beyond a controlled re-administration of the solution. Despite these limitations, this report aims to raise awareness among clinicians about the possibility of allergic reactions to commonly used crystalloids, emphasizing the need for vigilance to prevent adverse outcomes during medical treatments and also prompt identification and management including cessation of suspected agent and supportive care.

## CONCLUSION

This case illustrates that perioperative anaphylaxis can also be attributable to Ringer’s lactate. Unexplained intraoperative hypotension and cutaneous signs may be triggered by intravenous fluids. Early identification of the cause, cessation of exposure, and good supportive care are hence crucial for patient’s safety.
